# Identifying N6-Methyladenosine Sites in HepG2 Cell Lines Using Oxford Nanopore Technology

**DOI:** 10.3390/ijms242216477

**Published:** 2023-11-18

**Authors:** Viktoriia A. Arzumanian, Ilya Y. Kurbatov, Konstantin G. Ptitsyn, Svetlana A. Khmeleva, Leonid K. Kurbatov, Sergey P. Radko, Ekaterina V. Poverennaya

**Affiliations:** Institute of Biomedical Chemistry, Russian Academy of Medical Sciences, 119121 Moscow, Russia; viktoriia.arzumanian@ibmc.msk.ru (V.A.A.); ikurbatov@ibmc.msk.ru (I.Y.K.); konstantin157@yandex.ru (K.G.P.); diny1204@yandex.ru (S.A.K.); leonid15@mail.ru (L.K.K.); radkos@yandex.ru (S.P.R.)

**Keywords:** epitranscriptome, mRNA modifications, N6-methyladenosine, HepG2

## Abstract

RNA modifications, particularly N6-methyladenosine (m6A), are pivotal regulators of RNA functionality and cellular processes. We analyzed m6A modifications by employing Oxford Nanopore technology and the m6Anet algorithm, focusing on the HepG2 cell line. We identified 3968 potential m6A modification sites in 2851 transcripts, corresponding to 1396 genes. A gene functional analysis revealed the active involvement of m6A-modified genes in ubiquitination, transcription regulation, and protein folding processes, aligning with the known role of m6A modifications in histone ubiquitination in cancer. To ensure data robustness, we assessed reproducibility across technical replicates. This study underscores the importance of evaluating algorithmic reproducibility, especially in supervised learning. Furthermore, we examined correlations between transcriptomic, translatomic, and proteomic levels. A strong transcriptomic–translatomic correlation was observed. In conclusion, our study deepens our understanding of m6A modifications’ multifaceted impacts on cellular processes and underscores the importance of addressing reproducibility concerns in analytical approaches.

## 1. Introduction

RNA modifications, also known as epitranscriptomic modifications, play a crucial role in regulating RNA function and cellular processes. Among the over 300 identified RNA modifications, N6-methyladenosine (m6A) stands out as the most prevalent and well-researched modification in eukaryotic mRNA [1,2]. Notably, m6A greatly affects various RNA metabolic activities, including but not limited to splicing, translation, and decay [3]. Specifically, studies have highlighted that m6A, when present in the coding regions of human cells, particularly at the first position of the codon, acts as an inhibitor of translation [4]. This modification’s impact on translation can be multifaceted, influencing it in both positive and negative ways [5]. In the context of oncology, the m6A modification is pivotal in certain cancers where it supports the rapid translation of oncogenic proteins [6]. Moreover, disruptions in the m6A modification landscape are also associated with various pathological conditions, including but not limited to autoimmune [7] and cardiovascular diseases [8].

The identification of RNA modifications via conventional approaches, such as immunoprecipitation techniques (MeRIP-Seq, m6A-Seq, PA-m6A-Seq, etc.), enzyme-dependent methods (Mazter-Seq, m6A-REF-Seq, and DART-Seq), or chemical detection methods (Pseudo-Seq and AlkAniline-Seq), is associated with several limitations. While these techniques facilitate the generation of detailed transcriptome-wide maps of RNA modification sites, their applicability is constrained by the accessibility of antibodies or chemical agents specific to particular modifications. Furthermore, all these methodologies necessitate the reverse transcription and sequencing of cDNA followed by short-read sequencing to identify modifications. Yet, during each of these phases, a fraction of modifications may be lost. Additionally, these approaches lack single-nucleotide resolution and cannot identify modifications for single RNA molecules [9,10].

Oxford Nanopore technology (ONT) provides a novel approach to directly sequence RNA molecules without converting them to cDNA or utilizing PCR amplification. Direct RNA-Seq concludes the RNA sequence from the current intensity as RNA molecules traverse through the nanopores. Modified nucleotides generate a distinct signal intensity compared to their unmodified counterparts, enabling the computational determination of modification sites on each RNA molecule using either supervised (miCLIP [11], m6Anet [12], etc.) or comparative (Tombo [13], DRUMMER [14], Nanocompore [15], etc.) strategies. Comparisons of modification detection methods using only a single sample have shown that the m6Anet is more accurate than others [16]. This approach employs a multiple instance learning (MIL)-based neural network model, which leverages signal intensity and sequence attributes to pinpoint potential m6A sites in direct RNA-Seq data [12].

In this study, we employ the advanced capabilities of Oxford Nanopore technology to delve into the intricate world of m6A modifications, specifically targeting the HepG2 cell line. HepG2 cells stand out as a human liver cancer cell line and have been consistently adopted as a pivotal model system in extensive research endeavors that explore liver biology and associated diseases. A deeper and more nuanced understanding of the m6A modification patterns within these cell lines offers invaluable insights. These insights not only shed light on the intricacies of their involvement in liver cancer but also open up avenues for the potential conceptualization and development of innovative therapeutic strategies tailored to combat liver ailments.

Furthermore, to ensure the reliability and consistency of our findings, we conducted checks on the reproducibility of our results across technical repeats. This additional layer of scrutiny reiterates our commitment to ensuring that our research outputs are accurate, laying a solid foundation for future studies and applications.

## 2. Results

### 2.1. Detection of m6A Modification Sites

The analysis of five replicates for the HepG2 cell line detected 5173 modification sites, of which 3968 possible m6A modification sites occurred in protein-coding transcripts and 1205 sites occurred in non-coding ones. In a further analysis, we used sites in 2851 protein-coding transcripts corresponding to 1396 genes (Supplementary Table S1). The distribution among the replicates is presented in Table 1. As expected, when the amount of data obtained is small, the number of detected m6A modification sites is also low, except for the fourth replicate. Despite similar total information metrics between the second and fourth replicates, the number of sites in the latter is almost twice as high. This variation can be explained by the number of reads after the Basecalling and mapping steps. In the case of the second replicate, 1,175,355 reads remained and were further analyzed, while in the case of the fourth replicate, 1,630,381 reads were retained.

**Figure 1:**
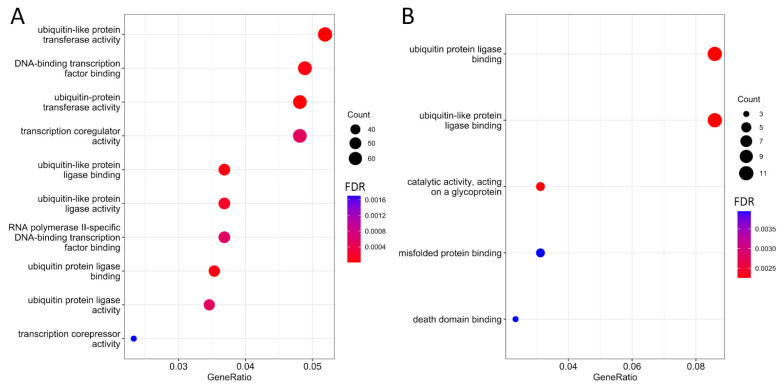
Molecular functions of m6A-modified sites within the transcript of a particular gene in the HepG2 cell line; (**A**) detected in all five technical replicates (*n* = 1396); (**B**) common to all five technical replicates (*n* = 132).

The genes in which possible m6A sites were predicted in the HepG2 cell line are involved in ubiquitination processes (Figure 1A), including “ubiquitin protein ligase binding” (FDR < 0.001), “ubiquitin-like protein ligase binding” (FDR < 0.001), “ubiquitin-protein transferase activity” (FDR < 0.001), and “ubiquitin-like protein transferase activity” (FDR < 0.001). This aligns with previous research on the role of m6A modifications, which has shown them to be critical regulators of histone ubiquitination events in cancers [17].

Additionally, these genes are involved in processes related to the regulation of transcription factors, including “RNA polymerase II-specific DNA-binding transcription factor binding” (FDR < 0.001) and “DNA-binding transcription factor binding” (FDR < 0.001). It has also been established that these genes participate in processes associated with “modification-dependent protein binding” (FDR < 0.001), indicating their involvement in protein binding during post-translational modification of target proteins [18].

### 2.2. Reproducibility between Technical Repeats

**Figure 2:**
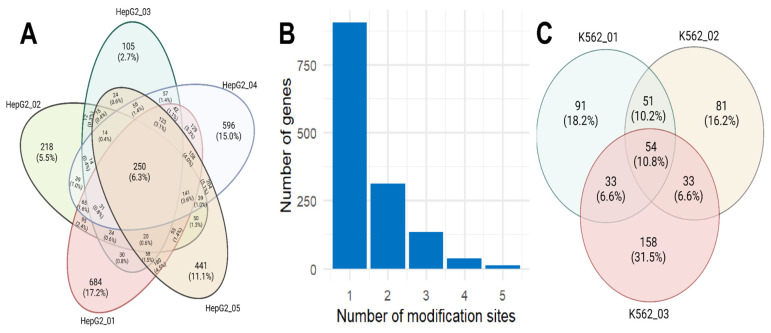
The distribution of detected m6A modification sites. (**A**) Overlap among the five technical replicates for the HepG2 cell line; (**B**) histogram of the number of m6A sites in protein-coding genes for the HepG2 cell line; (**C**) overlap among three technical replicates for the K562 cell line (public data were downloaded from Singapore Nanopore Expression Project [20]).

Since the data we previously obtained are technical replicates of direct RNA-seq performed using Oxford Nanopore technology on the same HepG2 sample, as with gene expression, we expected a high level of reproducibility in m6A modifications [19]. However, only 6.3% (250 out of 3968) of the possible m6A sites were detected in all replicates (Figure 2A). These sites correspond to 228 transcripts (8%), encoded by 106 (7.6%) genes. In nearly half of the cases of possible m6A modification sites (corresponding to 58% of the genes), they were identified only once. It is worth noting that the highest number of single occurrences were in the first (684 m6A sites) and fourth (596) replicates, while in large file sizes obtained in the second (2.3 Gb) and fifth replicates (2.6 Gb), the number of unique identifications was 218 and 441 m6A sites, respectively.

It is noteworthy that genes for which possible m6A sites were common for all technical replicates maintained associations with ubiquitination processes, such as “ubiquitin protein ligase binding” (FDR = 0.002) and “ubiquitin-like protein ligase binding” (FDR = 0.003) (Figure 1B), as well as protein binding processes, including “misfolded protein binding” (FDR = 0.004) and “death domain binding” (FDR = 0.004) (Figure 1B).

The majority (64.7%) of m6A sites are unique to each gene, with a maximum number of five modified sites per gene, an average of 1.5, and a median of 1 (Figure 2B). It is worth noting that genes with single sites often participate in protein binding processes like “modification-dependent protein binding” (FDR < 0.01), “methylated histone binding” (FDR < 0.01), and “methylation-dependent protein binding” (FDR < 0.01). Conversely, genes with multiple potential m6A sites are associated with ubiquitination processes such as “ubiquitin protein ligase binding” (FDR = 0.002) and “ubiquitin-like protein ligase binding” (FDR = 0.002).

These findings underscore the need to validate and improve algorithms for detecting modifications from a single sample, especially considering potential quality variations associated with Oxford Nanopore technology limitations. Nevertheless, we observe the preservation of functional trends, allowing us to integrate all detected potential m6A modification sites into further analysis.

To understand the reproducibility of the m6Anet method, we analyzed published direct RNA-seq data for the K562 cell line performed in three replicates within the Singapore Nanopore Expression Project (available online at https://github.com/GoekeLab/sg-nex-data, accessed on 29 October 2023) [20]. Only 10.8% (54 out of 501) of the potential m6A modification sites were consistently identified across all replicates (Figure 2B). These sites correspond to 49 transcripts (12%) encoded by 45 (12.7%) genes. In nearly half of the cases of possible m6A modification sites, they were identified only once.

It is important to emphasize that the number of detected modification sites in the published data was lower than in our studies. It is not possible to definitively say whether this is due to biological characteristics, or if the differences are caused by the varying volume of data in our and the published studies. It should be noted that the size of fastq files in the published data was around half that of ours. 

### 2.3. Impact of m6A Modifications on Gene Signatures

It is known that m6A modifications can enhance and inhibit translation [5]. Previously, for the studied HepG2 cell line sample, proteomic profiling was performed [21]. Additionally, translatome profiling was performed using the PolySeq method with Oxford Nanopore technology (see the Section 4).

In total, at the transcriptomic level, we detected 13,649 expressed genes in the HepG2 cell line (TPM > 0), confirmed at the translatomic level with 11,959 genes (TPM > 0) and at the proteomic level with 1027 genes (NSAF > 0). Among these, genes with potential m6A modification sites amounted to 1350 out of 1396 at the translatomic level and only 84 at the proteomic level.

**Figure 3:**
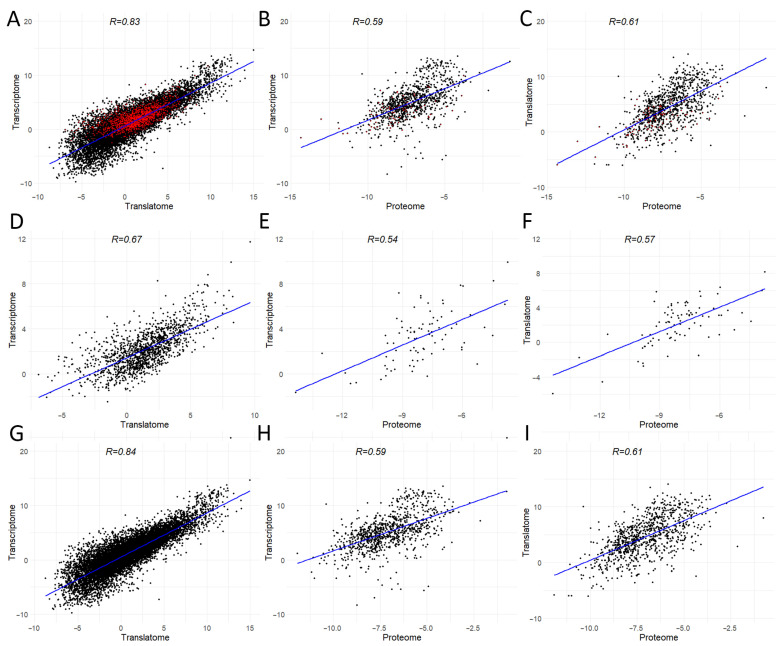
The correlation plots between transcriptomic, translatomic, and proteomic levels are presented in the following: (**A**–**C**) Correlation between all genes across the three levels of genetic information expression. Genes in the transcripts of which m6A modification sites were detected are highlighted in red. (**D**–**F**) Correlation between all genes with m6A modification sites across the three levels. (**G**–**I**) Correlation between all genes except those in the transcripts of which m6A modifications were detected. Expression levels are displayed in logarithmic values: log2(TPM) for transcriptome and translatome and log2(NSAF) for proteome.

We compared the correlation between different levels of genomic information expression for all detected genes (Figure 3A–C), separately for genes in the transcripts in which m6A modifications were detected (Figure 3D–F), and for all genes without considering the influence of genes in the transcripts in which m6A modifications were detected (Figure 3G–I).

In the case of HepG2 cells, when comparing all detected genes, the highest correlation was observed between the transcriptome and translatome (R = 0.83), while the lowest correlation was found between the transcriptome and proteome (R = 0.59) (Figure 3A–C). For genes in the transcripts of which m6A modifications were detected, a similar trend was observed, with the Spearman correlation values for transcriptome–translatome (Figure 3D), transcriptome–proteome (Figure 3E), and translatome–proteome (Figure 3F) being lower than those observed for all genes combined (Figure 3A–C). Most identified potential m6A modification sites appear to reduce the translation of their corresponding genes, as evidenced by the correlations between genes without considering these 1396 modified genes, where the Spearman correlation value for transcriptome–translatome is the highest among all comparisons (Figure 3G), while for transcriptome–proteome and translatome–proteome, the correlation remains unchanged (Figure 3H,I, respectively) compared to all genes.

The existing analytical limitations in proteomic methods prevented us from identifying specific features of genetic information expression for all 1369 genes with detected m6A modifications. However, the even distribution of expression for these genes at the transcriptomic and translatomic levels suggests that the patterns observed for the 84 genes at the proteomic level can be generalized. In particular, this assertion will be valid, at the very least, for genes associated with protein binding processes (Supplementary Figure S1).

## 3. Discussion

Our analysis aimed to identify m6A modification sites in the HepG2 cell line. The limiting factor in this analysis for identifying m6A modification sites is the low reproducibility of results between technical sequencing replicates. It should be noted that published studies using the m6Anet algorithm did not emphasize the issue of reproducibility of identifications between technical replicates. However, data reproducibility seems to be an important aspect when using supervised learning algorithms [12]. In our view, the obtained results are primarily influenced by the characteristics of the Oxford Nanopore method, where due to the biological nature of the method, it is impossible to obtain the same volume and quality of RNA sequencing data for one sample on two chips. This fact may be disregarded for transcriptomic analysis, where algorithmic solutions are robust to minor discrepancies, but for supervised learning algorithms, this is crucial.

Within the framework of our study, we also relied on published data to assess the reproducibility of our results. The analysis revealed limited correspondence between the results of three technical replicates. Furthermore, a smaller number of modified sites was recorded in the K562 cell line (chronic myelogenous leukemia) compared to the HepG2 line (hepatoblastoma). However, it is important to note that such differences may be caused not only by the characteristics of these cell lines but also by the difference in the volume of the initial data used.

Given the issues that have arisen, we recommend using aggregate information from multiple technical replicates to enhance the reliability of m6A modification site identification. Looking forward, addressing reproducibility issues remains a priority.

**Figure 4:**
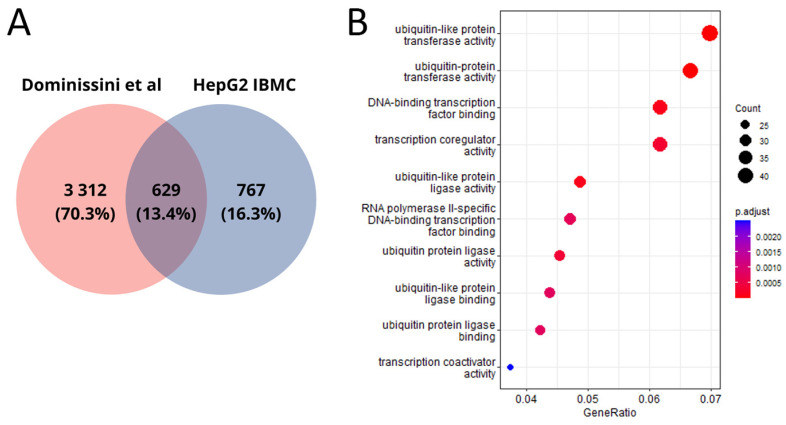
(**A**) Venn diagram between public data [10] and our HepG2 genes; (**B**) molecular functions of genes in the transcripts where m6A modification sites that are common between our and public data were detected. As in other studies on m6A analysis [17], we observed a significant enrichment of genes for which m6A modifications were predicted with ubiquitylation processes. Ubiquitylation is a form of post-translation modification that plays a critical role in maintaining cellular homeostasis and regulating numerous biological processes, including the cell cycle [23,24]. Disruption of ubiquitylation contributes to the development of pathological processes, mainly tumors [24,25].

Our work is not the first in the context of detecting m6A modification sites in the HepG2 cell line. Given the dependence of the algorithm used on the training dataset, we did not expect identical results. Overall, we identified half as many genes with m6A modifications as in the work by Dominissini et al. [10], where the m6A-seq method was used for m6A modification detection. Only 13.4% of genes with potential m6A modification sites were identified in both studies (Figure 4A). The common genes are also associated with ubiquitination processes (Figure 4B).

Differences in results can be explained by methodological aspects. The training dataset for the m6Anet algorithm was trained on results obtained using the m6ACE method, while the published data were obtained using the m6A-seq method. Traditional sequencing methods are known to face issues such as a high level of false positives, insufficient reproducibility, and low resolution [22].

Based on the fact that functional trends are preserved for genes with identified m6A modification sites, in the case of multiple technical replicates, we recommend using the cumulative information obtained from them.

We observed that the majority of the potential m6A modification sites we identified are associated with decreased translation of the corresponding genes. However, there is evidence that m6A may also enhance translation [5]. The impact of these modifications depends on multiple factors, including the presence of specific m6A reader proteins and the location of m6A on the mRNA [5,26,27,28].

It is also worth mentioning that the limitations of proteomic methods prevented us from fully examining the impact of m6A modifications on the translation of all transcripts. Nevertheless, the uniform distribution of gene expression possessing m6A modification sites at the transcriptomic and translatomic levels suggests that the trends we observed at the proteomic level could be more widely applicable.

## 4. Materials and Methods

### 4.1. Translatome Profiling

The HepG2 cell line was obtained from Sigma-Aldrich (Merck KGaA, Darmstadt, Germany), and cells were cultured as previously described [19] until they reached ~80% confluence. Prior to harvesting, cells were incubated for 5 min in the presence of cycloheximide (Merck, 100 µg/mL). The harvested cells were washed twice with phosphate-buffered saline (supplemented with 100 µg/mL of cycloheximide) and were counted, and 10 million cells were pelleted using centrifugation. The pellet was resuspended in 500 µL of the lysing buffer (20 mM Tris-HCl, pH 7.4, 130 mM NaCl, 10 mM Mg_2_Cl, 1% CHAPS, 0.02% heparin, 2.5 mM DTT, 100 µg/mL of cycloheximide, 40 U/mL of SUPERase·In, 50 U/mL of Turbo DNase I, and 20 µL of Complete Protease inhibitor). The suspension was passed at least 5 times through a 23 G needle with a syringe and put on ice for 10 min to complete lysis. The lysate was centrifuged for 10 min at 20,000× *g* (4 °C), and the supernatant was transferred to a new test tube. This operation was repeated twice. Approximately 350 μL of the supernatant was finally collected and put on ice for further use.

Since it is commonly accepted to profile the translatome and transcriptome in parallel (e.g., [29,30]), we used the same cell lysate for both translatome and transcriptome sequencing. A total of 350 μL of lysate supernatant was divided into two parts and used for isolating total RNA (50 μL) and for the chromatographic fractionation to obtain polysomes (~300 μL).

Total RNA was isolated with the RNA Clean & ConcentratorTM-25 kit (Zymo Research, Irvine, CA USA), according to the manufacturer’s instructions. Polysomes were isolated using the Ribo Mega-SEC method [31] based on size-exclusion chromatography, closely following the protocol described in [32]. An aliquot of the HepG2 cell lysate (100 µL) was loaded onto a precooled (4 °C) Agilent Bio SEC-5 column (5 µm, 2000 A, 7.8 × 300 mm; Agilent, Santa Clara, CA USA) equilibrated with the run buffer (20 mM Tris-HCl, pH 7.4, 60 mM NaCl, 10 mM Mg_2_Cl, 0.3% CHAPS, 0.01% heparin, and 2.5 mM DTT). The fractionation was performed on an Agilent 1100 Series chromatograph at 4 °C with a flow rate of 0.8 mL/min. The representative chromatogram is shown in Supplementary Figure S2. The 0.3 mL fractions of 2 to 6 (Supplementary Figure S2), containing polysomes [31,32], were pooled and concentrated to 50 µL on a Vivaspin-2 30 K cut-off concentrator (Sartorius, Edgewood, NY, USA). To this concentrate, 5 µL of 10% SDS was added, and RNA was isolated with the RNA Clean & ConcentratorTM-25 kit. Both total RNA and polysome RNA were quantified on a Qubit fluorometer (Thermo Fisher Scientific, Waltham, MA, USA) with the Qubit RNA HS Assay kit (Thermo Fisher Scientific) and stored at −80 °C until sequencing library preparation. The RNA quality was assessed using the 2100 Bioanalyzer system (Agilent)—the RIN values were 7.8 or above.

The preparation of sequencing libraries was carried out with the PCR-cDNA Sequencing kit (SQK-PCS111, ONT, Oxford, UK), strictly following the manufacturer’s protocol. The 14 cycles of PCR were conducted for both total RNA and polysome RNA. The long-read sequencing was carried out on a MinION nanopore sequencer (ONT) in 48 h single runs, using FLO-MIN106 flow cells.

The raw sequencing data for the HepG2 translatome were deposited in the NCBI SRA (https://www.ncbi.nlm.nih.gov, accessed on 1 September 2023) under accession number PRJNA972889.

### 4.2. Data Analysis

**Figure 5:**
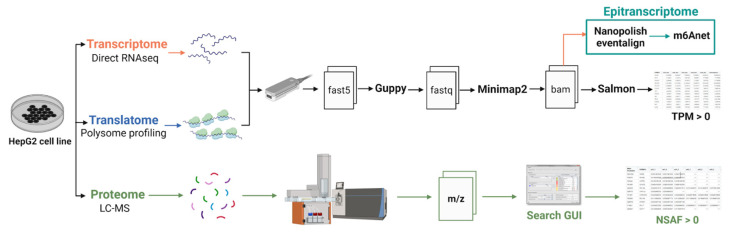
The overall workflow of the experiment used for the detection of m6A RNA modifications.

The transcriptome and proteome data were described in Pytnitskiy et al. [19] and Poverennaya et al. [21], respectively. We utilized data from the same HepG2 cell line. The raw sequencing data for the HepG2 transcriptome were deposited in the NCBI SRA (https://www.ncbi.nlm.nih.gov, accessed on 1 September 2023) under accession number PRJNA765908. The raw proteome data were uploaded to Mendeley Data (https://data.mendeley.com/, accessed on 1 September 2023). Data for the K562 cell lines were obtained from the Singapore Nanopore Expression Project (https://github.com/GoekeLab/sg-nex-data, accessed on 29 October 2023) [20]. The overall workflow of the experiment, during which m6A RNA modifications were discovered and gene expression was detected at the transcriptomic, translatomic, and proteomic levels, is presented in Figure 5. Transcriptome and translatome profiling were analyzed using the same protocol. Basecalling was performed using Guppy (version 6.2.1). The resulting fastq files were aligned using the long-read aligner Minimap2 (“-ax map-ont” mode, version 2.24) [33]. Gene expression and isoform analysis were performed using Salmon [34]. Expression of each transcript was quantified in transcript per million (TPM) units, giving relative abundance. Gene expression was calculated by summing all the TPM values of corresponding transcripts.

We used Nanopolish (version 0.14.0) for the resquiggling step [35]. To identify RNA modifications from direct RNAseq data, we used m6Anet (version 2.0.2) [12]. We used the default human model trained on the HCT116 cell line. Modification sites with a modification ratio less than 0.9 were excluded from our analysis. In our manuscript, we have adopted the term “possible m6A modification sites” in alignment with the terminology used by the authors of the m6Anet paper, who refer to such sites as “predicted” rather than “detected”. We converted raw proteome data into mgf format using MSConvert (v. 3) [36]. For proteome data processing, the SearchGUI software (v. 4.1.24) was employed, leveraging search engines such as X!Tandem, MS-GF+, and OMMSA, and matched against the SwissProt library containing both canonical and alternatively spliced human protein sequences in its automatic mode [37]. We set a false discovery rate (FDR) threshold of ≤1% for peptide–spectrum matches, peptides, and proteins. For label-free quantification in proteomics, we utilized the normalized spectral abundance factor (NSAF) method [38].

The R software environment was used for computations and visualization (ver. 4.1) [39]. We used ClusterProfiler [40] for over-representation analysis. To enrich our analysis with biological knowledge, we leveraged Gene Ontology (GO), specifically focusing on the molecular function (MF) orthogonal ontology [41].

## 5. Conclusions

In conclusion, our analysis has highlighted the active involvement of genes with detected m6A modification sites in critical cellular processes, particularly in ubiquitination, aligning with prior research linking m6A modification to histone ubiquitination in cancer. Additionally, these genes play roles in regulating transcription factors and protein folding processes.

Furthermore, our examination of various expression levels, including the transcriptomic, translatomic, and proteomic levels, revealed a robust correlation between the transcriptomic and translatomic levels. However, the correlation with the proteomic level was slightly lower, underscoring the intricacies involved in translating mRNA levels into functional proteins and other regulatory processes.

Our study sheds light on the intricate interplay between technical nuances and biological functions in epitranscriptomic research. It underscores the need for methodological rigor and highlights the importance of considering both technical and biological factors in the identification of m6A modification sites.

## Figures and Tables

**Table 1:** Number of predicted modifications in HepG2 cell line.

Repeats	Number of Possible m6A Modification Sites	Number of Corresponding Protein-Coding Transcripts	Number of Corresponding Protein-Coding Genes	Number of Expressed Protein-Coding Transcripts	Total Yield (Gb)
HepG2_1	2085	1659	814	36,001	2.9
HepG2_2	1082	908	462	34,898	2.3
HepG2_3	874	743	383	31,078	1.4
HepG2_4	1957	1556	792	34,341	2.2
HepG2_5	1827	1467	710	34,517	2.6

## Data Availability

All raw files were uploaded to the NCBI SRA (https://www.ncbi.nlm.nih.gov), accession PRJNA765908 for transcriptoime and PRJNA972889 for translatome data. Raw proteome data are publicly released on the Mendeley Data (https://data.mendeley.com/), DOI: 10.17632/yrmd8ygncn.1.

## Supplementary Materials

The following supporting information can be downloaded at https://www.mdpi.com/article/10.3390/ijms242216477/s1.
