# Supplementary figures and images for: Identifying N6-Methyladenosine Sites in HepG2 Cell Lines Using Oxford Nanopore Technology
Source: Int J Mol Sci. 2023 Nov 18;24(22):16477. doi: 10.3390/ijms242216477 (PMC10671286; doi:10.3390/ijms242216477)

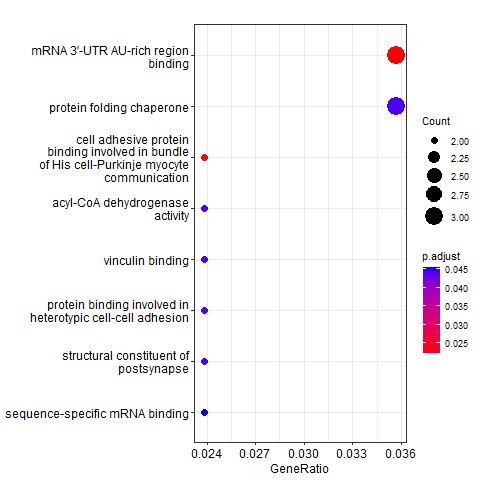

Supplement: Supplementary file 1 [file ijms-24-16477-s001.zip › Figure S1. GO analysis for 84 genes at the proteomic level.png]

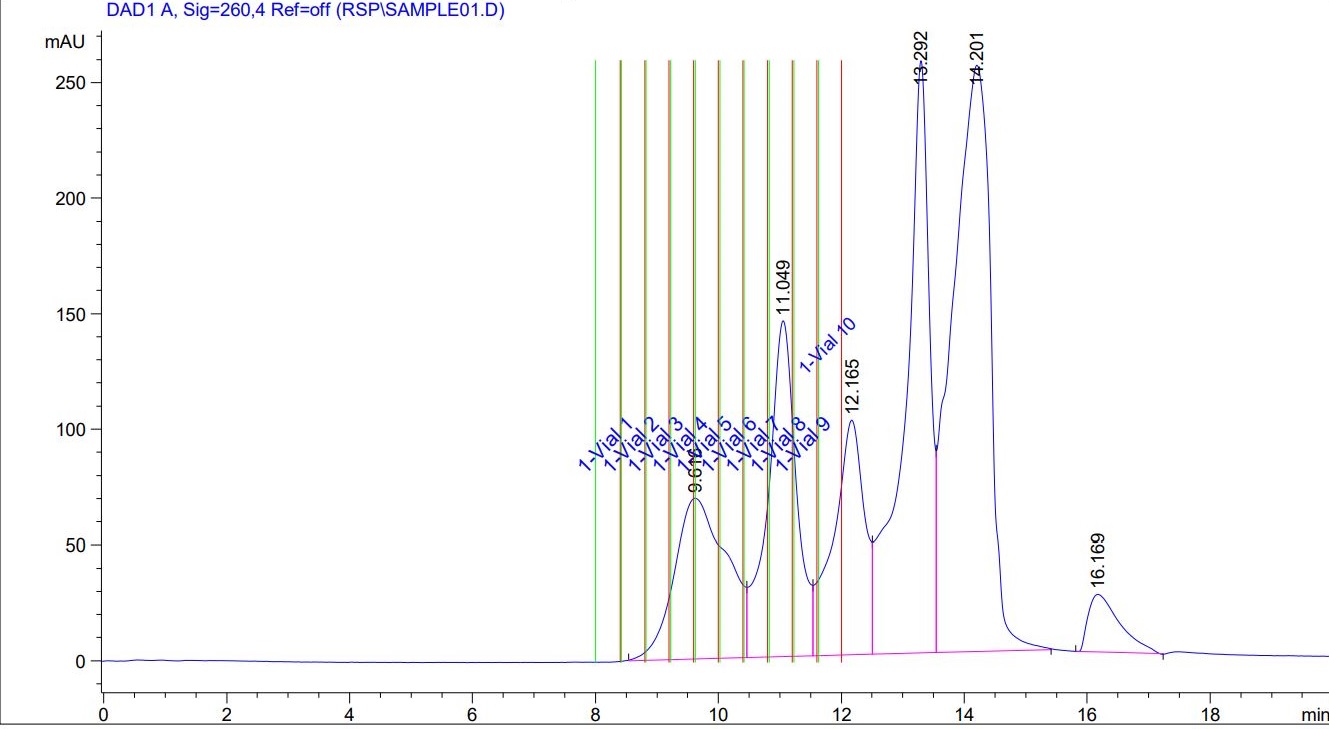

Supplement: Supplementary file 1 [file ijms-24-16477-s001.zip › Figure S2. chromatogram.JPG]
